# Effects of feed form and feed particle size with dietary L- threonine supplementation on performance, carcass characteristics and blood biochemical parameters of broiler chickens

**DOI:** 10.1186/2055-0391-56-20

**Published:** 2014-09-25

**Authors:** Vahid Rezaeipour, Sepideh Gazani

**Affiliations:** Department of Animal Science, Qaemshahr Branch, Islamic Azad University, PO Box 163, Qaemshahr, Iran

**Keywords:** L-threonine, Feed form, Broiler, Performance

## Abstract

An experiment was conducted to evaluate the effect of form and particle size of feed supplemented with L- threonine on growth performance, carcass characteristic and blood biochemical parameters of broiler chickens. The experimental design was a 2 × 2 × 2 factorial arrangement of treatments evaluating two feed forms (pellet or mash), two feed particle sizes (fine or course), and two inclusion rates of dietary L-threonine (with or without) which adopted from 7 to 42 days of age. In this experiment, 360 a day old chicks in two sexes were assigned in each treatment and each experimental unit was included 15 chicks. Feed consumption and weight gain were measured weekly. At 35 days of age, blood samples were taken to analysis blood biochemical parameters. At the end of the experimental period, two birds were slaughtered in each treatment and carcass analysis was carried out. The results showed that the effect of feed form on body weight gain and feed intake in whole of experimental period was significant (*P* < 0.05). Broilers fed pelleted diets had more weight gain than the mash group. Growth performance parameters were not affected by feed particle size and dietary L-threonine supplementation in whole of experimental period (*P* > 0.05). The results of carcass analysis showed that liver and gizzard relative weights were influenced by feed form (*P* < 0.05). However, pancreas and liver relative weights were affected by feed particle size and dietary L-threonine supplementation, respectively (*P* < 0.05). Triglyceride and VLDL levels were affected by feed form and dietary L-threonine supplementation (*P* < 0.05). The effect of feed particle size on blood biochemical parameters was not significant (*P* > 0.05). In conclusion, the experimental results indicated that feed form increased feed consumption and weight gain in whole of experimental period (1 to 42 days of age) while feed particle size and dietary L-threonine had no effect on broiler performance.

## Background

Interest in the effects of feed form and particle size has increased in recent years in Iran. Therefore, some of the poultry feed industry members search for ways to optimize the utilization of feed and improve poultry production. Particle size reduction increases both the number of particles and the surface area per unit volume allowing greater access to digestive enzymes and easier mixing of the ingredients [1]. Recommendations regarding optimum particle size, however, have been contradictory, as the results from feeding trials are confounded by a number of factors including feed physical form, complexity of the diet, grain type, endosperm hardness, grinding method, pellet quality and particle size distribution [2].

Different types of feed form have been evolved in broiler production at the present time. Mash is a form of a complete feed that is finely ground and mixed so that birds cannot easily separate out ingredients. Pellet system of feeding is really a modification of the mash system. It consists of mechanically pressing the mash into hard dry pellets. The greatest advantage in using pellets is that there is little waste in feeding. It is suggested that the large-grain particle size influenced broiler performance to a greater extent when birds were fed mash diets than when fed pelleted or crumbled diets [3, 4].

Threonine is the third most limiting amino acid, especially in a low crude protein diet. L-threonine is added to the diet of pigs and poultry in order to exactly match the dietary amino acid balance with the unique nutritional requirements of the animal [5]. Dozier et al. [6] indicated that broilers fed inadequate threonine had decreased live performance, but no effects were apparent on carcass fat. It is reported that supplementation of L-threonine as a source of dietary-threonine in combination with *Saccharomyces cerevisiae* improved growth performance and intestinal morphology traits in broilers [5]. Therefore, it is necessary to balance threonine in broiler diets by adding L-threonine supplementation or use of soybean meal and meat meal as most important ingredients which supply threonine in the chick diets.

Therefore, the objectives of this study were to investigate the effects of feed form and particle size with L-threonine supplementation on performance, carcass characteristics and blood biochemical parameters of broilers.

## Methods

### Birds and diets

Three hundred and sixty 1 day-old Ross 308 broiler chicks (Mixed sex) with initial body weight of 46 ± 2 g were obtained from a local hatchery (Chalak Jojeh, Qaemshahr, Iran). Birds were weighed and randomly assigned to 24 floor pens with 15 birds per pen so that the average weight per pen was similar. Average initial body weight of chicks was 46 ± 3 g. The chicks were raised on floor pens for 42d and had free access to feed and water during the whole period of experiment. The ambient temperature was maintained at 32°C for the first 3d and then gradually decreased until 24°C was reached by 21d. Experimental procedures were approved by Islamic Azad University, Qaemshahr branch.

Corn grain was ground in hammer mill to achieve a size of 3 or 6 mm for fine and course grades, respectively. Half of each formulated diet was fed in mash form and other half was cold pelleted with a pellet mill. Dietary L-threonine supplementation was added to each starter and grower diets. The ingredients profile and chemical composition of dietary treatments are shown in Table 1.

**Table 1:** **The ingredients and chemical composition of basal diets**

	Basal diets		
Ingredients	Starter		Grower
		(g/kg)	
Corn grain	624.0		645.0
Soybean meal	322.0		292.0
Corn oil	15.0		27.0
DCP	19.0		18.0
Limestone	5.0		4.0
Common salt	2.5		2.1
Mineral premix^1^	5.0		5.0
Vitamin premix^1^	5.0		5.0
DL-Methionine	2.5		2.0
L-Lysine	1.8		1.3
L-Threonine	0.6		0.4
Sodium bicarbonate	1.2		2.0
Salinomycine	0.5		0.5
**Calculated analysis (%)**			
AME (kcal/kg)	3000		3100
Crude protein	19.5		18.00
Calcium	0.90		0.90
Phosphorous, available	0.45		0.45
Met + Cys	0.88		0.88
Lysine	1.15		1.12

^1^Provides per kg of diet: 9000 I.U. vitamin A; 2000 I.U. vitamin D3; 18 I.U. vitamin E; 2 mg menadion; 1.8 mg thiamine; 6.6 mg riboflavin; 30 mg niacin; 3 mg pyridoxine; 15 mg vitamin B12; 100 mg D-pantothenic acid; 1 mg folic acid; 0.1 mg biotin; 500 mg choline chloride; 100 mg antioxidant; 100 mg manganese; 84.7 mg zinc; 50 mg iron; 10 mg copper; 1 mg iodine; 0.2 mg selenium.

### Experimental procedure and sampling

All birds were group weighed by pen after overnight fasting at 21 and 42d. Feed intake on group basis was measured at the same time intervals. Feed conversion ratio for each pen was calculated by dividing feed intake to body weight gain. Mortality was checked daily and weighed for adjusting feed conversion ratio.

Blood sampling of two birds per each pen was done at 35 d of age. Samples were left for three hours at room temperature to coagulate the blood and then Serum was isolated by centrifugation (3000 g, 15 min) to blood chemistry measurements. Serum Triglyceride, total cholesterol and glucose were analyzed using enzymatic colorimetric kits (Pars Azemon, Iran).

At the end of the experiment (on day 42), after overnight fasting , two birds from each pen (four birds per each treatment) with a body weight close to pen mean were selected and killed. Viscera were manually removed and carcass characteristics were determined. Then, empty weight of total digestive tract, caeca, gizzard, liver (without gallbladder) and pancreas were measured. All carcass data are presented based on percent of live weight of each bird.

### Statistical analysis

The experiment was conducted using completely randomized design with factorial structure. Pen mean was an experimental unit. Effect of feed particle size (fine or course), feed form (pellet or mash), and dietary L-threonine (with or without) were statistically analyzed as 2 × 2 × 2 factorial design by General Linear Models (GLM) procedure of SAS [7]. Probability values <0.05 were taken to indicate statistical significance using Duncan multiple range test.

## Results

The results of growth performance of birds are shown in Table 2. The results showed that weight gain and feed intake were not affected by particle size and L-threonine supplementation in whole of experimental period (*P* >0.05). However, pelleted form and L-threonine supplementation increased body weight gain of broilers at 1–21 days of age (*P* <0.05). The birds fed with pellet form had a better weight gain than those fed with mash form in whole of experimental period. The results showed that feed consumption increased in birds fed pelleted diets at all phases of growth period. The results also revealed that the birds fed diets without L-threonine supplementation had better weight gain (1-21d) and feed intake (21-42d) than those fed with L-threonine supplementation. Effects of all dietary treatments on feed conversion ratio were not statistically significant (*P* >0.05).

**Table 2:** **Effects of dietary treatments on growth performance (gr/day) of broilers**

Treatment	Weight gain			Feed intake			FCR ^1^		
	1-21	21-42	1-42	1-21	21-42	1-42	1-21	21-42	1-42
**Feed form**									
Mash	56.7^b^	66.0	61.4^b^	91.7^b^	121.9^b^	106.8^b^	1.61	1.86	1.74
Pellet	61.5^a^	70.4	65.9^a^	101.8^a^	131.8^a^	116.7^a^	1.65	1.89	1.77
**Feed size**									
Course	59.4	66.7	63.1	97.5	127.2	112.0	1.64	1.91	1.78
Fine	58.8	69.6	64.2	95.9	126.5	111.6	1.63	1.83	1.73
**L-threonine**									
+	60.3^a^	66.9	63.6	97.3	124.6^b^	110.9	1.61	1.88	1.75
-	57.9^b^	69.5	63.7	96.2	129.2^a^	112.7	1.66	1.86	1.76
SEM	0.69	1.79	0.89	0.61	1.36	0.90	0.02	0.04	0.02
**Interaction effects**									
	Probability value								
A × B	0.67	0.93	0.94	0.20	0.71	0.86	0.78	0.87	0.93
A × C	0.56	0.10	0.15	0.01	0.57	0.16	0.34	0.03	0.01
B × C	0.24	0.17	0.35	0.17	0.71	0.83	0.60	0.20	0.25
A × B × C	0.25	0.10	0.04	0.69	0.66	0.63	0.34	0.09	0.03

^a-b^Means without a common superscripts in per column are significantly different (P < 0.05).

^**1**^FCR: Feed conversion ratio.

A: feed form; B: feed size; C: L-threonine.

The results of carcass parameters (Table 3) showed that except for liver and gizzard relative weights, none of carcass parameters affected by dietary treatments (*P* >0.05). The results of Table 3 indicated that liver relative weight was greater in birds fed pelleted diet without L-threonine supplementation. There was no significant difference for pancreas relative weight in birds fed course or fine diets. Gizzard relative weight increased in birds fed mash diets.

**Table 3:** **Effects of dietary treatments on carcass characteristics and internal organs of broilers**

Treatment	Breast	Thigh	Liver	Pancreas	Gizzard	Heart	Intestine
	(% of live weight)						(cm)
**Feed form**							
Mash	25.55	18.76	2.22^b^	0.21	1.19^a^	0.42	211.62
Pellet	26.64	18.25	2.44^a^	0.20	0.99^b^	0.44	215.66
**Feed size**							
Course	25.92	18.49	2.26	0.19	1.10	0.44	217.12
Fine	26.27	18.52	2.39	0.21	1.09	0.42	210.16
**L-threonine**							
+	26.04	18.49	2.22^b^	0.20	1.06	0.43	215.62
-	26.15	18.52	2.44^a^	0.21	1.12	0.43	211.66
SEM	0.38	0.23	0.06	0.01	0.03	0.01	2.96
**Interaction effects**							
	Probability value						
A × B	0.44	0.18	0.51	0.61	0.79	0.48	0.30
A × C	0.03	0.14	0.08	0.15	0.65	0.24	0.001
B × C	0.33	0.59	0.23	0.38	0.77	0.08	0.21
A × B × C	0.14	0.43	0.16	0.49	0.30	0.32	0.06

^a-b^Means without a common superscripts in per column are significantly different (P < 0.05).

A: feed form; B: feed size; C: L-threonine.

The results of blood biochemical parameters are shown in Table 4. Results showed that Glucose, cholesterol, HDL and LDL concentrations did not affect by dietary treatments (*P* >0.05). Triglyceride and VLDL concentration increased in birds fed pellet diets without L-threonine supplementation.

**Table 4:** **Effects of dietary treatments on blood biochemical characteristics of broilers**

Treatment	Cholesterol	Triglyceride	Glucose	LDL	HDL	VLDL
	(mg/dl)					
**Feed form**						
Mash	128.8	94.7^b^	232.9	67.1	43.2	18.9^b^
Pellet	129.5	133.9^a^	258.8	68.8	41.5	26.6^a^
**Feed size**						
Course	137.7	119.2	259.6	68.7	43.6	23.7
Fine	120.6	109.4	232.5	67.2	41.0	21.8
**L-threonine**						
+	125.6	87.7^b^	237.3	68.2	42.7	17.5^b^
-	132.7	140.9^a^	254.5	67.6	42.0	28.1^a^
SEM	7.96	9.80	16.03	2.33	1.44	1.98
**Interaction effects**						
	Probability value					
A × B	0.53	0.38	0.96	0.44	0.77	0.37
A × C	0.10	1.00	0.83	0.06	0.15	0.97
B × C	0.45	0.68	0.60	0.86	0.60	0.66
A × B × C	0.41	0.13	0.80	0.67	0.84	0.12

^a-b^Means without a common superscripts in per column are significantly different (P < 0.05).

A: feed form; B: feed size; C: L-threonine.

## Discussion

Growth performance was not affected by particle size in present study. The effects of particle size on broiler performance have been studied in a number of researches [8]. The results of Behnke [8] indicated that particle size increased the surface area of the grain thus allowing for greater interaction with digestive enzymes [8]. Most researchers suggested that reducing mean particle size of cereal grains results in marked improvements in nutrient digestibility and efficiency of growth [9]. In contrast Kilburn and Edwards [10] suggested that large particle size of soybean meal in diets of broilers was more efficiently than fine particle size. Possibly these larger particles of soybean meal passed through the digestive tract at a slower rate and improved the nutrient utilization.

Interaction between particle size and feed physical form in broiler diets for weight gain and feed intake is well documented [4, 11, 12]. Svihus et al. [11] reported that feeding pelleted diets increased weight gain and feed intake and improved the feed conversion ratio compared with those birds maintained on mash diets. The results of present study support these findings. Amerah et al. [4] indicated that it may be due to increased nutritional density, improved starch digestibility resulting from chemical changes during pelleting, increased nutrient intake, changes in the physical form of the feed, reduced feed wastage, and decreased energy expenditure in eating. Nir et al. [3] reported that broilers fed wheat and sorghum mash diets with coarser particles had heavier body weights and better feed efficiency compared to those fed the finely ground diets.

The digestive tract development of poultry in known to be affected by feed particle size. Gabriel et al. [13] and Nir et al. [3] reported that relative intestinal weight was lower in birds fed coarse particle diets compared to those fed fine particle diets. The present data showed that the relative gizzard weight were higher in birds fed mash feeds than in those fed pelleted feeds. Similar findings have been reported by Nir et al. [3] and Amerah et al. [4]. These results may suggest that pelleting decreased the grinding requirement of the gizzard so that its function was reduced to that of transit.

Effect of L-threonine supplementation on broiler performance was not significant in present study. It is demonstrated that formulation of diets to contain up to 544 g of L-threonine/ton did not affect growth or carcass attributes of commercial broilers. It is well known that use of L-threonine decreased protein requirements of broilers and improved nitrogen efficiency utilization [14]. Rezaeipour et al. [5] indicated that dietary L-Threonine improved intestinal morphology and growth rate in broilers.

A dearth of information exists in terms of blood parameters in poultry; therefore, direct comparison cannot be made.

## Conclusions

In conclusion, the data showed that feed form and dietary L-threonine had a greater influence on the different measured parameters than did the particle size. In summary, pellet form increased feed consumption and weight gain in whole of experimental period (1 to 42 days of age) while feed particle size and dietary L-threonine had no effect on broiler performance at the same time.

## References

[1] Goodband RD, Tokach MD, Nelssen JL (2002). MF-2050 Feed Manufacturing.

[2] Amerah AM, Ravindran V, Lentle RG, Thomas DG (2007). Influence of feed particle size and feed form on the performance, energy utilization, digestive tract development, and digesta parameters of broiler starters. Poult Sci.

[3] Nir I, Hillel R, Ptichi I, Shefet G (1995). Effect of particle size on performance. 3. Grinding pelleting interaction. Poult Sci.

[4] Amerah AM, Ravindran V, Lentle RG, Thomas DG (2008). Influence of feed particle size on the performance, energy utilization, digestive tract development, and digesta parameters of broiler starters fed wheat- and corn- based diets. Poult Sci.

[5] Rezaeipour V, Fononi H, Irani M (2012). The effects of dietary L-threonine and *Saccharomyces cerevisiae* on performance, intestinal morphology and immune response of broiler chickens. S Afr J Anim Sci.

[6] Dozier WA, Moran ET, Kidd MT (2000). Threonine requirement of broiler males from 42 to 56 days in a summer environment. J Appl Poult Res.

[7] *SAS, Statistical Analysis Systems User's Guide, Version 8.02 Edition*. Cary, N.C. USA: SAS Institute, Inc; 2001.

[8] Behnke KC (1996). Feed manufacturing technology: current issues and challenges. Anim Feed Sci Technol.

[9] Ebrahimi R, Bojar pour M, Mokhtarzadeh S (2010). Effect of feed particle size on the performance and carcass characteristics of broilers. Int J Anim Vet Adv.

[10] Kilburn J, Edwards HM (2001). The response of broiler to the feeding of mash or pelleted diets containing maize of varying particle sizes. Br Poult Sci.

[11] Svihus B, Klovstad KH, Perez V, Zimonja O, Sahlstrom S, Schuller RB (2004). Physical and nutritional effects of pelleting of broiler chicken diets made from wheat ground to different coarsenesses by the use of roller mill and hammer mill. Anim Feed Sci Technol.

[12] Peron A, Bastianelli D, Oury FX, Gomez J, Carre B (2005). Effects of food deprivation and particle size of ground wheat on digestibility of food components in broilers fed on a pelleted diet. Br Poult Sci.

[13] Gabriel I, Mallet S, Leconte M (2003). Differences in the digestive tract characteristics of broiler chickens fed on complete diet or on whole wheat added to pelleted protein concentrate. Br Poult Sci.

[14] Kidd MT, Kerr BJ (1997). Threonine responses in commercial broilers at 30 to 42 days. J Appl Poult Res.

